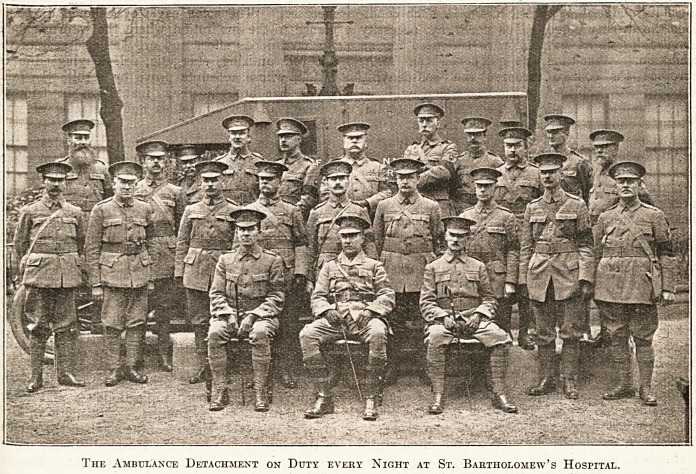# Ambulance Detachment on Duty Every Night at St. Bartholomew's Hospital

**Published:** 1915-12-04

**Authors:** 


					December 4, 1915 THE HOSPITAL 203
THE CTTY 0"F LONDON NATIONAL GUARD.
The Ambulance Detachment on Duty every Night at St. Bartholomew's Hospital.
.
.  1
The Ambulance Detachment on Duty every Night at St. Bartholomew's Hospital.

				

## Figures and Tables

**Figure f1:**